# Investigating Psychometrics of Career Decision Ambiguity Tolerance Scale

**DOI:** 10.3389/fpsyg.2019.02067

**Published:** 2019-09-10

**Authors:** In-Jo Park, Shenyang Hai, Seungmi Lee, Youngwoo Sohn

**Affiliations:** ^1^Department of Psychology, Henan University, Kaifeng, China; ^2^Department of Psychology, Yonsei University, Seoul, South Korea

**Keywords:** career decision, career decision ambiguity tolerance, career indecision, career decision self-efficacy, tolerance

## Abstract

This study aimed to validate career decision ambiguity tolerance scale-Korean form applicable to a Korean sample. In study 1, 17-items from the original 18-item career decision ambiguity tolerance scale were valid based on IRT. In study 2, using the confirmatory factor analysis, we showed that excluding item 4 from the original scale is better than including it in the three factors model. Given the results of study 1 and 2, the constructs in the 17-item career decision ambiguity tolerance scale-Korean form were valid. In study 3, career decision ambiguity tolerance positively predicted career decision-making self-efficacy, career indecision, and career adaptability, respectively, after controlling for calling and career search self-efficacy. Thus, the incremental validity of the career decision ambiguity tolerance scale-Korean form was ensured. In study 4, the reliability of the scale was retained as the test-retest (conducted over a 4-week period) demonstrated adequate results.

## Introduction

In the twenty first century, it is a worldwide phenomenon for individuals to have difficulty in career decisions because they have difficulty collecting information to fit an appropriate career into a rapidly changing global environment and instability of economy. Korea is one such country, where companies are laying off senior employees and are stagnant in hiring new employees (Kim and Yoon, 2015). Individuals are confronted with career decision difficulty and should tolerate ambiguity in terms of determining their career (Xu and Tracey, 2014a). Tolerance for career decision ambiguity appears to be one of the most important career issues for both college students and employees considering turnover or seeking a second career after retiring. In this vein, it is necessary for psychologists and career counselors to detect and evaluate the degree to which individuals can tolerate ambiguity in their career decision process. Accordingly, Xu and Tracey (2015) conducted research in which they developed and validated a career decision ambiguity tolerance scale.

Ambiguity tolerance is a personality variable, reflecting an individual's emotional and perceptual aspect (Frenkel-Brunswik, 1949). Budner (1962) defined ambiguity tolerance as “the tendency to perceive ambiguous situations as desirable.” It influences reactions to a spectrum of situational features suitable in various life contexts (Furnham and Marks, 2013). Empirical research showed that ambiguity tolerance is related to better performance, willingness to violate ethical norms, and consumers' willingness to pay for remanufactured products (Teoh and Foo, 1997; Weisbrod, 2009; Hazen et al., 2012). On the other hand, intolerance of ambiguity is associated with challenge appraisal, life satisfaction, and positive affect (Bardi et al., 2009). Regarding career behaviors, ambiguity tolerance predicts career decision-making self-efficacy (Xu and Tracey, 2014a) and indirectly predicts career indecision via environmental and self-exploration (Xu and Tracey, 2014b).

Xu and Tracey (2015) recently adapted the ambiguity tolerance construct and introduced the idea of career decision ambiguity tolerance (CDAT) to career research. Based on Budner's (1962) tripartite model of ambiguity tolerance which consisted of three constructs, namely tolerance toward unfamiliarity, complexity, and inconsistent information, they defined CDAT as “*people's evaluations of and responses to unfamiliar, complex, or inconsistent information in career decision making”* (Xu and Tracey, 2015).

CDAT would be frequently utilized to explore individuals' career decision process. Accordingly, the CDAT scale, which was developed and validated by Xu and Tracey (2015), consists of three subscales containing 18 statements in total for three subscales. These three subscales are *preference, tolerance*, and *aversion*, in which each factor contains 6 statements. This original scale's validity and reliability were ensured through exploratory and confirmatory factor analysis, incremental validity, and test-retest reliability. However, though cross-cultural validation studies can greatly benefit career researchers and career counselors, no research has been conducted cross-culturally to extend the CDAT scale's validity in Korea. For instance, validating the Korean CDAT scale could allow researchers to investigate its role on career decision and to provide career counselors with a sound psychometric tool for evaluating their Korean clients.

We are thus interested in the Mokken and Rasch rating models in the Item Response Theory Analysis (IRT) for validating the CDAT scale-Korean form in this study. The IRT is useful for investigating scale validity because it provides psychometric characteristics with many indices (e.g., scalability, item difficulty, and point measure correlation). The Mokken model was originally used to analyze dichotomous data, and a model of polytomous data in a Likert scale was subsequently introduced (Molenaar, 1982). The Mokken model assesses whether each item evaluates the same underlying concept. The scalability of a scale is evaluated by Loevinger's coefficient *H*, which compares the actual Guttman errors to the items' expected errors (Crichton, 1999; Sijtsma et al., 2011). Also, existing scales are validated with psychometric evaluation using the Rasch rating model (Park et al., 2015). The Rasch rating model was developed for polytomous items. This model assesses whether the difference between any given threshold location for items and the mean of the threshold locations are coherent across items (Wright et al., 1994). The Rasch rating model reports several indices including item difficulty, point measure correlation, and difference item function, which is useful for confirming scales' construct validity.

The current study aimed to validate the CDAT scale-Korean form. To validate this scale, we first attempted to evaluate the psychometric properties for each CDAT scale item using the Rasch rating and Mokken models. Second, confirmatory factor analysis was performed to compare model fit between the different two models. Third, we explored incremental validation through hierarchical regression analysis. Lastly, a test-retest was conducted with 4-weeks' delay in order to confirm the CDAT scale-Korean form's reliability.

## Study 1: Item Response Theory Analysis

Study 1 aimed to validate the CDAT scale-Korean form using the Mokken and the Rasch rating models. The Mokken model reports indices confirming the scalability of each item. The Rasch model provides indices estimating item difficulty and data fit relative to the model. These two models are useful for ensuring the scale's construct validity.

### Methods

#### Participants and Procedures

In total, 323 participants were part of this study. All participants were attending psychology courses from 7 universities, including Calvin, Chonnam, Choonsing Cyber, Dankuk, Gwandong, and Gyeonsang Universities in South Korea. Furthermore, 87 (26.9%) were male and 236 (73.1%) were female. The mean age was 31.64 (*SD* = 11.91), though ages ranged from 19 to 64 years.

Surveys were administered after the authors explained the current study's goals. We received consent forms from all participants in this study. The participants accessed a website (www.surveymonkey.com) using their smartphone or laptop computer to respond to the survey questions. If a participant did not respond to any question, he or she could not finish the survey. The duration including the study's explanation and the questionnaire response time was about 7 min. Participants received course credits if they completed the entire survey questionnaire.

This study was conducted in accordance with the Declaration of Helsinki and was approved by Yonsei University's review board. All participants gave written informed consent in accordance with the Declaration of Helsinki.

#### Instrument

##### The career decision ambiguity tolerance scale

The CDAT scale (Xu and Tracey, 2015) is an 18-item measure designed to assess an individual's evaluation and response to the career decision process, based on the Budner's tripartite model (Budner, 1962). The CDAT scale consists of three domains, namely preference (e.g., “It is interesting to discover new strengths and weakness”), tolerance (e.g., “I am tolerant of the potential difference between my perception and the reality of a career”), and aversion (e.g., “I find difficult to make career decisions as things cannot be predicted clearly”); each domain, respectively contained 6-items. Following the translation guidelines of Beaton et al. (2000), the authors used forward-backward and counter-translation to translate the CDAT scale into Korean for the current study. Participants were asked to rate a 7-point Likert scale, which ranged from 1 (*strongly disagree*) to 7 (*strongly agree*). Xu and Tracey (2015) found a Cronbach's alpha of 0.79, 0.69, and 0.83, respectively for the preference, tolerance, and aversion factors. This study also showed a Cronbach's alpha of 0.79, 0.76, and 0.83, respectively for the preference, tolerance, and aversion factors.

#### Analyses

In the current study, we performed the Mokken model analyses using MSPWIN5 (Molenaar et al., 2000a). The Mokken model assumes there is an underlying continuum applied to the scale's items (Schmitz et al., 2000). In this study, the preference, tolerance, and aversion subscales were considered as underlying, latent constructs. This model reports a scale coefficient, Loevinger's *H*, to ensure the scalability for both the individual scale items and the scale itself. An *H* value should be ≥0.3 for it to be an acceptable value for determining the scalability for each item and the overall scale (Carter et al., 2015).

Rasch model analyses were conducted using WINSTEPS 3.81.0 for Windows (Linacre, 2014). Data fit, item difficulty, and response category relevance were calculated using the Rating Scale Model (RSM; Wright and Masters, 1982). In the RSM, the ability of the individual indicates the measured psychological trait's strength, and item difficulty indicates the trait strength required for individuals to typically agree with an item. The RSM is designed for polytomous items, which are effective in validating short versions of scales.

### Results

Loevinger's *H* for all items is provided in Table 1. In the current study, item 4 (i.e., “I am not interested in knowing new information about myself”) had weak scalability (*H* = 0.27). The H values of all other items were ≥0.3, which indicated that the other 17-items have acceptable scalability. The overall scale *H* for preference, tolerance, and aversion, respectively, were 0.41, 0.37, and 0.47, which was acceptable as subscale. *Rho* was used as scale reliability; an estimated value above 0.70 is considered to indicate a reliable scale (Molenaar et al., 2000b). The subscales of preference (*Rho* = 0.74), tolerance (*Rho* = 0.78), and aversion (*Rho* = 0.83) all had good reliability.

**Table 1:** *H*-coefficients for CDAT-Korean form items (*N* = 323).

**Item**	***H***	**Mean**
**Preference**		
1. It is interesting to discover new strengths and weaknesses	0.49	5.65
2. I am interested in exploring the many aspects of my personality and interests	0.43	5.63
3. I am excited to see a creative way to match my interests with a career	0.50	5.76
4. I am not interested in knowing new information about myself	0.27	5.81
5. I am excited that I can learn new things about myself or about the world when making a career decision	0.48	5.54
6. I am open to careers which I have never heard of or thought of before	0.32	5.20
**Tolerance**		
7. I do not mind changing my career in the future if necessary	0.42	4.92
8. I am tolerant with the possibility that my interests could change in the future	0.47	5.40
9. I am tolerant of the unpredictability of a career	0.36	5.44
10. I enjoy tackling complex career decision making tasks	0.30	4.38
11. I am tolerant of the potential difference between my perception and the reality of a career	0.38	5.26
12. I am able to make a choice when multiple options seem equally appealing	0.32	4.51
**Aversion**		
13. People's different or sometimes contradictory perspectives about a career make me uncomfortable	0.31	3.85
14. I try to avoid complicated career decision making tasks	0.46	3.72
15. The career decision making process, which involves so many considerations, is just daunting	0.55	4.07
16. I find it difficult to make career decisions as things cannot be predicted clearly	0.58	4.35
17. I try to avoid a career in which the prospects cannot be foreseen clearly	0.40	4.33
18. I am afraid of sorting out the complex aspects of a career	0.55	3.98

Unidimensionality was examined through principal component analysis of residuals (PCA). Principal component analysis is used to verify the Rasch model. Because the Rasch model requires unidimensionality, principal component analysis is used to test whether a substantial factor exists in the residuals after the main measurement dimension has been estimated (Smith, 2002). Linacre (2014) suggested that the eigenvalue of unexplained variance explained by the first contrast should be below 20%. In this study, we separately conducted the Rasch model with each factor (i.e., preference, tolerance, and aversion factors). The obtained eigenvalue of unexplained variance explained by the first contrast for the preference, tolerance, and aversion factors, respectively, was 14.2, 18.4, and 12.7%; hence, the data met Linacre's criterion.

The absence of redundant items and item homogeneity can be confirmed by fit statistics (Linacre and Wright, 1994). Fit indices are calculated by comparing obtained responses of an item with the expected score generated by the Rasch model. The expected score expresses the item's construct validity and possible responses (Bond and Fox, 2007). Table 2 shows Infit and Outfit statistics for all 18 CDAT scale items. If the Infit and Outfit values of an item equal 1.0, the item perfectly fits within the expected score. If the fit value is below 0.5 or above 1.5, the item is not validly useable. Item 4 violated this criterion as it showed 1.67 and 1.66, respectively, for Infit and Outfit. The other 17-items for fit indices varied from 0.66 to 1.48; hence, 17-items met the Infit and Outfit criterion [i.e., 0.5 ≤ MNSQ (mean square residual) ≤ 1.5].

**Table 2:** Statistics of Rasch model for 323 participants.

	**MNSQ**				**Point-measure**
**Scale item**	**Infit**	**Outfit**	**Item difficulty**	**S.E**.	**Correlations**
1	0.66	0.71	−0.06	0.07	0.70
2	1.02	1.02	−0.02	0.07	0.66
3	0.72	0.72	−0.22	0.07	0.71
4	1.67	1.66	−0.29	0.07	0.53
5	0.75	0.76	0.10	0.06	0.72
6	1.28	1.39	0.50	0.06	0.60
7	1.01	1.05	0.09	0.06	0.69
8	0.74	0.71	−0.46	0.06	0.72
9	1.03	1.02	−0.46	0.06	0.69
10	1.10	1.11	−0.50	0.05	0.62
11	0.80	0.80	−0.46	0.06	0.64
12	1.09	1.19	0.51	0.50	0.61
13	1.41	1.48	0.20	0.06	0.56
14	1.02	1.06	0.34	0.06	0.69
15	0.74	0.73	−0.02	0.06	0.79
16	0.66	0.63	−0.31	0.06	0.82
17	1.47	1.43	−0.29	0.06	0.66
18	0.70	0.72	0.07	0.06	0.78

*Item difficulty, item difficulty estimate. MNSQ, mean square residuals. S.E., standard error*.

Item difficulty estimates and item characteristic curves (ICCs) were obtained for all 18-items. Item difficulty estimates for all items ranged from −0.50 to 0.51. Seven items returned item difficulty estimates of >0 (more difficult items) and the other 11 items returned difficulty estimates of <0 (easier items) (see Table 1). Item 10, with an item difficulty estimate of −0.50, was the easiest item; item 12, with an item difficulty estimate of 0.51, was the most difficult.

We also verified the CDAT scale's content validity. Content validity expresses whether the items evaluate all aspects of the construct they were intended to assess (Bryant, 2000). Point measure correlations are used to assess the items' content validity (Guiberson and Rodriguez, 2014). The point measure correlation value, ranging from −1 to 1, should be ≥0.3 to ensure adequate content validity (Bond and Fox, 2007; Linacre, 2014). In our study, the point measure correlation values of all items were above 0.30 (see Table 2).

## Study 2: Confirmatory Factor Analysis

In study 1, item 4's scalability and fit indices failed to ensure its validity. The authors wondered whether the structural factor by excluding item 4 from the CDAT scale would exacerbate the validity of this scale. The purpose of study 2 was to compare one model (i.e., inclusion of item 4) with the other (i.e., exclusion of item 4) using confirmatory factor analysis.

### Methods

#### Participants and Procedures

In total, 224 participants were recruited for this study. All participants were attending psychology courses in Gumho, Gyeongsang, Kunkuk, and Sogang Universities; 125 (55.8%) were male and 99 (44.2%) were female. The mean age was 22.59 (*SD* = 2.31) and ranged from 19 to 35 years.

The procedure of study 2 was the same as study 1 (i.e., based on online survey). The total duration of the survey was about 7 min.

#### Analyses

We conducted a confirmatory factor analysis using AMOS 20.0 with the maximum likelihood estimation method. The Chi-square, comparative fit index (CFI), root mean square error of approximation (RMSEA), and Akaike information criterion (AIC) were employed to explore suitable data representation between the two models (Jöreskog, 1983; Hu and Bentler, 1999). The AIC is especially useful in this study because the AIC estimates the quality of each model relative to other models (Akaike, 1987). Hair et al. (2010) suggested that values of CFI > 0.90 and RMSEA <0.08 indicate an acceptable model fit. A difference in AIC value between two models (ΔAIC) > 2 indicates the significance of the difference (Jöreskog, 1983).

### Results

Based on the original CDAT's factor structure, we performed a confirmatory factor analysis (CFA) with a three-factor oblique model. The RMSEA value was 0.08, which is acceptable (Hu and Bentler, 1999). The CFI value was 0.85, which was comparable to the original CDAT scale's value of 0.86 (Xu and Tracey, 2015).

Next, we eliminated item 4 from the three-factor oblique model and then performed a CFA; this is because study 1 reported that item 4 had poor values for item scalability, Infit, and Outfit. The results showed that the values for RMSEA (0.08) and CFI (0.85) were the same as the previous analysis with 18-items (see Table 3). To select the appropriate model, we compared the two models using AIC values. The 3-factor with 17-items had an AIC = 388.12 while the 3-factor with 18-items resulted in AIC = 421.71; the difference between them was *Δ*AIC = 33.59. If ΔAIC is above 2, the difference is considered significant (Jöreskog, 1983). Accordingly, we endorsed the 3-factor with 17-items as the representative structure for the Korean CDAT scale.

**Table 3:** Summary of model fit indices for CFA models.

					**RMSEA**	
	**χ^2^**	**df**	**AIC**	**CFI**	**Estimates**	**90% C.I**.
3-factor with 17 items	280.12	116	388.12	0.85	0.08	[0.068, 0.092]
3-factor with 18 items	307.71	132	421.71	0.85	0.08	[0.066, 0.089]

## Study 3: Incremental Validity

In this study, we aimed to validate the CDAT scale-Korean form in order to widely apply this concept to the Korean population. Xu and Tracey (2015) showed that the CDAT was associated with constructs in the career decision process, which included career decision-making self-efficacy, career indecision, and career adapt-abilities. Previous studies also found that career search self-efficacy is related to career decision-making self-efficacy, career indecision, and career adapt-abilities (Solberg et al., 1995; Nota et al., 2007). Given these previous studies, we explored CDAT's influences on career decision processes like career decision-making self-efficacy, career indecision, and career adapt-abilities, even after controlling for career search self-efficacy. In this way, the CDAT scale-Korean form's incremental validity was examined in the current study.

### Methods

#### Participants and Procedures

In total, 213 participants were recruited for this study. All participants were attending an architecture course at Gumho University in South Korea; 154 (72.3%) were male and 59 (27.7%) were female. The mean age was 23.21 (*SD* = 2.29), though ages ranged from 19 to 36 years.

The procedure of study 3 was the same as studies 1 and 2 (i.e., using online surveys). The total duration of the survey was about 15 min.

#### Instruments

In this study, all concepts were used by combing the different domains of these scales. Scale scores were calculated by the average of all items of the scale such that higher scores indicate greater levels of the concept.

##### Career decision-making self-efficacy-short form (CDSE-SF)

Career decision-making self-efficacy was measured using a 25-item Career Decision-Making Self-Efficacy Scale-Short Form (CDSE-SF) developed by Betz and Voyten (1997). The CDSE-SF consists of five domains, including (a) self-appraisal (e.g., “Figure out what you are and are not ready to sacrifice for your career goals”), (b) occupational information (e.g., “Find the average yearly earnings of people in an occupation”), (c) goal selection (e.g., “Make a career decision and then not worry if it was right or wrong”), (d) planning (e.g., “Determine the steps you need to successfully complete your chosen major”), and (e) problem solving (e.g., “Persistently work at your major or career goal even when you get frustrated”). Participants were asked to rate their confidence in each item using a 5-point Likert scale, which ranged from 1 (*no confidence at all*) to 5 (*complete confidence*). Lee (2001) translated the CDSE-SF into Korean and utilized it in his study. Cronbach's alpha for this scale was 0.92 in Lee's (2001) study and 0.91 in the current study.

##### Career adapt-abilities scale-korean form (CAAS)

The CAAS-Korean form assesses an individual's career adapt-abilities. Savickas and Porfeli (2012) initially developed and validated the CAAS-USA form, which contained 24-items. The CAAS-USA form consists of four subscales with 6-items each to assess (a) concern (e.g., “Thinking about what my future will be like”), (b) control (e.g., “Making decision by myself”), (c) curiosity (e.g., “Becoming curious about new opportunities”), and (d) confidence (e.g., “Taking care to do things well”). Respondents were required to rate each item on a 5-point Likert scale, which ranged from 1 (*not strong*) to 5 (*strongest*). Tak (2012) translated the CAAS-USA form into Korean and subsequently validated it. Cronbach's alpha was 0.92 in Savickas and Porfeli (2012) study and 0.93 in Tak (2012) study. This study found a Cronbach's alpha of 0.74.

##### Career indecision

Career indecision was assessed with the Career Decision Scale (CDS) subscale developed by Osipow et al. (1976), an 18-item subscale that was translated into Korean for our study. Example items include “I have decided on a career and feel comfortable with it. I also know how to go about implementing my choice” and “Several careers have equal appeal to me. I am having a difficult time deciding among them.” Each item in the scale was rated on a 5-point Likert scale, which ranged from 1 (*like me*) to 5 (*not like me*). The test-retest reliabilities with a 2-week delay (0.82 and 0.90) were recorded in the test manual (Osipow, 1980). The Cronbach's alpha was 0.91 in this study.

##### Career search self-efficacy

Career search self-efficacy was evaluated using the Career Search Self-Efficacy (CSSE) Scale developed by Solberg et al. (1994). The items were described with the leading phrase “How confident are you in ability to…” An example item includes “Identify and evaluate your career values.” Respondents in the original CSSE scale were required to rate the items on a 10-point scale ranging from 0 (*very unconfident*) to 9 (*very confident*). Choi (2006) validated the CSSE-Korean form with a 5-point Likert scale, which ranged from 1 (*very unconfident*) to 5 (*very confident*). Cronbach's alpha of this scale was 0.94 in the current study.

##### Career decision ambiguity tolerance

We used the CDAT scale-Korean form with 17-items in this study. This study reported a Cronbach's alpha of 0.78, 0.69, and 0.82, respectively, for the preference, tolerance, and aversion factors.

#### Analyses

We performed Harman's single factor test to assess common method bias (Harman, 1967). If significant common method bias exists in the measurement, factor analysis of all items would generate a single factor that explains most of the variance. Because the first factor of the unrotated solution explained 28.59% of the variance in the data, we concluded that common method variance is not a major concern in this study.

We performed a hierarchical regression analysis using SPSS 20.0 to examine the CDAT scale-Korean form's incremental validity. In step 1, career search self-efficacy was entered as the baseline predictor for three criteria sets including career decision-making self-efficacy, career indecision, and career adapt-abilities. We also entered age and gender as control variables because previous studies have suggested that age and gender were associated with career decision making self-efficacy and career adapt-abilities (Gianakos, 2001; Scott and Ciani, 2008; Zacher, 2014). In step 2, we entered the different factors (i.e., “preference,” “tolerance,” and “aversion”) of the CDAT scale-Korean form.

### Results

Having provided evidence of incremental validity, we sought to determine if the CDAT scale-Korean form could associate with relevant outcomes such as career decision-making self-efficacy, career indecision, and career adaptability; even after controlling for career search self-efficacy, age, and gender, which were associated with these outcomes. As such, a series of hierarchical regression analyses were conducted (Cohen et al., 1983). Descriptive statistics and inter-correlations for all variables are provided in Table 4.

**Table 4:** Descriptive statistics and inter-correlations for all variables (*N* = 213).

	**Mean**	**SD**	**1**	**2**	**3**	**4**	**5**	**6**	**7**	**8**
1. Age	23.21	2.29								
2. Gender	1.28	0.45	−0.28^**^							
3. CSSE	3.36	0.66	0.14^*^	−0.15^*^						
4. CDSE-SF	3.46	0.53	0.19^**^	−0.12	0.84^**^					
5. CI	2.97	0.70	−0.17^*^	0.11	−0.43^**^	−0.45^**^				
6. CAAS	3.77	0.57	0.19^**^	−0.19^**^	0.76^**^	0.73^**^	−0.40^**^			
7. Preference	5.43	0.89	0.07	−0.08	0.46^**^	0.48^**^	−0.19^**^	0.59^**^		
8. Tolerance	5.04	0.83	0.15^*^	−0.12	0.43^**^	0.50^**^	−0.17^**^	0.47^**^	0.65^**^	
9. Aversion	4.13	1.10	−0.16^*^	0.20^**^	−0.45^**^	−0.45^**^	0.57^**^	−0.40^**^	−0.24^**^	−0.31^**^

* *p < 0.05*,

** *p < 0.01. CSSE, career search self-efficacy; CDSE, career decision-making self-efficacy; CI, career indecision; CAAS, career adaptability*.

In the first series of equations, four control variables were entered in Step 1. In Step 2, CDAT scale-Korean form's three subscales that represent the three factors (i.e., preference, tolerance, and aversion) were entered. Results of the three hierarchical regression analyses are listed in Table 5. The preference, tolerance, and aversion factors predicted an additional 3% of the variance in career decision-making self-efficacy [Δ*F*_(3, 203)_ = 7.55, *p* < 0.001], an additional 18% of the variance in career indecision [Δ*F*_(3, 203)_ = 19.02, *p* < 0.001], and an additional 8% of the variance in career adaptability [Δ*F*_(3, 203)_ = 17.26, *p* < 0.001] above what was accounted for by the control variables. Tolerance was significantly associated with career decision-making self-efficacy (β = 0.15, *p* < 0.01); aversion was significantly associated with career indecision (β = 0.49, *p* < 0.001), while preference was significantly associated with career adaptability (β = 0.33, *p* < 0.001). Thus, CDAT scale-Korean form's incremental validity was ensured in this study.

**Table 5:** Hierarchical regression results (*N* = 213).

**Step**	**Variable**	β^*^	**SE**		***R^**2**^***	**Δ*F***
CDSE-SF						
Step 1	Age	0.02	0.01	0.08	0.71	159.08^***^
	Gender	0.02	0.05	0.02		
	CSSE	0.67	0.03	0.83^***^		
Step 2	Preference	0.03	0.03	0.05	0.74	7.55^***^
	Tolerance	0.10	0.03	0.15^**^		
	Aversion	−0.03	0.02	−0.06		
CI						
Step 1	Age	−0.03	0.02	−0.11	0.20	16.42^***^
	Gender	0.04	0.10	0.03		
	CSSE	−0.43	0.07	−0.41^***^		
Step 2	Preference	−0.04	0.06	−0.05	0.38	19.02^***^
	Tolerance	0.11	0.06	0.13		
	Aversion	0.31	0.04	0.49^***^		
CAAS						
Step 1	Age	0.02	0.01	0.07	0.59	93.85^***^
	Gender	−0.07	0.06	−0.05		
	CSSE	0.63	0.04	0.74^***^		
Step 2	Preference	0.21	0.04	0.33^***^	0.67	17.26^***^
	Tolerance	0.00	0.04	0.00		
	Aversion	−0.03	0.02	−0.05		

* *p < 0.05*,

** *p < 0.01*,

*** *p < 0.001. CDSE, career decision-making self-efficacy; CSSE, career search self-efficacy; CI, career indecision; CAAS, career adaptability*.

## Study 4: Test-Retest Reliability

Test-retest reliability was calculated to confirm the 17-item CDAT scale-Korean form's stability.

### Methods

#### Participants and Procedures

In total, 47 undergraduate students enrolled in an introductory psychology course at Gyeongsang University in South Korea participated in the first session: 17 (36.2%) were male and 30 (63.8%) were female. The mean age was 22.96 (*SD* = 1.93), though their ages ranged from 20 to 29 years.

This study asked the participants to rate the CDAT scale-Korean form two times over a 4-week interval. Among the 47 participants, 42 students attended the retest session. Each session took roughly 7 min from the study explanation to completing the responses.

#### Analyses

Using SPSS 20.0, we examined the test-retest reliability. The test-retest reliabilities of the three subscales were produced by calculating correlation coefficients over a 4-week interval.

### Results

In the first test, the Cronbach's alpha results were 0.78, 0.60, and 0.67, respectively, for the preference, tolerance, and aversion subscales. The retest showed a Cronbach's alpha of 0.78, 0.66, and 0.82, respectively, for preference, tolerance, and aversion. The test-retest reliability coefficients over the 4-week interval were 0.63, 0.69, and 0.66.

## Discussion

The current study attempted to validate the CDAT scale-Korean form's usefulness for future research in career behaviors. In study 1, the results showed that the CDAT scale-Korean form had the proper construct validity for all items except item 4 (i.e., “I am not interested in knowing new information about myself”). In study 2, based on the confirmatory factor analysis, the results showed that excluding item 4 is better than its inclusion in terms of model fit. Accordingly, we considered the 17-item CDAT scale-Korean form, in which item 4 was excluded from the original CDAT scale. In study 3, the CDAT positively associates with career decision-making self-efficacy, career indecision, and career adaptability after controlling for career search self-efficacy, age, and gender. Thus, the CDAT scale-Korean form's incremental validity was ensured through these processes. In study 4, the CDAT scale-Korean form's reliability was retained as the test-retest over a 4-week interval showed adequate correlation coefficients for the three subscales (i.e., “preference,” “tolerance,” and “aversion”). Given our studies' results, we suggest that researchers and career counselors may validly use the CDAT-Korean form in the future.

In study 1, except item 4, the results indicated that all 17-items reached the required criteria for scalability, Infit, Outfit, and point measure correlation (Mokken, 1971; Linacre and Wright, 1994; Bryant, 2000). In other words, item 4 did not obtain adequacy for those indices. Item 4 is the only reverse-worded item in the original CDAT scale, which could lead to responding error. We thus hypothesize several reasons why item 4 could not satisfy these indices' criteria. First, bias with reverse-worded item results from the respondent acquiescence effect whereby raters uncritically agree with the item (Ray, 1983). Raters would respond to the item in the same direction with previous items if they were familiar with the items. Second, respondent inattention is one of the reasons exacerbating construct validity (Schmitt and Stults, 1985). Namely, raters often carelessly respond to reverse-worded items. Third, reverse-worded items are difficult to understand. Swain et al. (2008) reported that the probability of false response is likely to increase with reverse-worded items because raters need more time to process them. Thus, even though reverse-worded items have some advantages like reducing social desirability (Hughes, 2009), they could generate response errors and thus compromise construct validity. In study 3, the results showed that construct validity improved when the reverse-worded item (item 4) was deleted from the scale. We therefore suggest that researchers use the 17-item CDAT-Korean form in future studies.

In study 3, we explored how CDAT associated with career decision-making self-efficacy, career indecision, and career adaptability; career search self-efficacy, age, and gender were controlled to ensure incremental validity. The results showed that the tolerance factor was associated with career decision-making self-efficacy, aversion was associated with career indecision, and preference was associated with career adaptability. These findings are interesting prediction patterns and have implications for career research and counseling. First, aligned to a prior study (Xu and Tracey, 2015), the association of the tolerance factor with career decision-making self-efficacy indicates that an individual's career decision-making self-efficacy is positively influenced by their CDAT tolerance. Practically, career counselors could enhance clients' career decision-making self-efficacy by helping them improve their career decision tolerance.

Second, the relationship between the aversion factor and career indecision indicates that individuals with high aversion for career decision are likely to report greater career indecision. Ambiguity aversion diminishes individuals' willingness to act under uncertainty (Fox and Weber, 2002); thus, aversion for career decision leads to career indecision. This result suggests that CDAT aversion is one of the most important factors in the career decision process as well as other factors such as anxiety (e.g., Fuqua et al., 1987), neuroticism (e.g., Page et al., 2008), and time perspective (e.g., Savickas et al., 1984) that have also been associated with career indecision.

Third, CDAT's preference factor is related to career adaptability. Given the concept of career adaptability by Savickas and Porfeli (2012), in which they described career adaptability as “denoting an individual's resources for coping with current and anticipated tasks, transition, trauma in their occupational roles that, to some degree large or small, alter their social integration” (Savickas, 1997), the preference factor would be considered as a facilitator that enhances mental resources in career decision processes.

The current study's findings support that the CDAT scale-Korean form is a culturally valid tool to assess the Korean population's career decision ambiguity tolerance. Xu and Tracey (2015) argued that few studies have been conducted for ambiguity tolerance in career behavior research, as a domain-specific measure in career decision was absent. In this vein, they developed and validated the original CDAT scale so that researchers can explore career behaviors in various contexts. Through our validation study of the CDAT scale-Korean form, researchers worldwide as well as in Korea can utilize this scale for future research on the Korean population. Practically speaking, the CDAT scale-Korean form is useful to career counselors in Korea because they can assess clients' ambiguity tolerance in their career decision domain, thus enabling counselors to guide or train their clients.

The current study has limitations that should be addressed in the future. The first limitation is that we used a survey in study 3 to examine the predictive validation that gave rise to common method bias and thus potentially threatens our conclusion's validity (Podsakoff et al., 2003). To ensure predictive validity for the CDAT scale-Korean form, future research should be conducted longitudinally. Second, participants in study 4 were asked to rate the CDAT scale-Korean form twice over a 4-week period to ensure the test-retest reliability that could have introduced carryover effect. We recommend future studies to extend this duration in order to strengthen the reliability of this scale. Third, George and Mallery (2003) suggested that above 0.7 for Cronbach's alpha ensures internal reliability for a scale. However, in the present study, the tolerance subscale has relatively low internal consistency reliability in study 3, wherein Cronbach's alpha was 0.69. In study 4, Cronbach' alpha for tolerance was 0.60 and Cronbach' alpha for aversion was 0.67 at T1 and Cronbach' alpha for tolerance was 0.66 at T2. The value of Cronbach's alpha is sensitive to number of participants, in which a greater number of participants generates a higher Cronbach' alpha value. The total number of participants is small in study 4, thereby showing a lower Cronbach's alpha value. However, future studies are required to address this issue with different samples. Lastly, similar to the original CDAT scale validation study (Xu and Tracey, 2015), we utilized an undergraduate population in all four studies. To strength the validity of the CDAT scale-Korean form, future studies should recruit working adults or younger students, thus allowing comparison with college students.

## Data Availability

All datasets for this study are included in the manuscript/supplementary files.

## Author Contributions

I-JP analyzed data and wrote paper. SH designed this study. SL collected the study. YS revised the paper.

### Conflict of Interest Statement

The authors declare that the research was conducted in the absence of any commercial or financial relationships that could be construed as a potential conflict of interest.
